# A Novel Technique for Conjunctivoplasty in a Rabbit Model: Platelet-Rich Fibrin Membrane Grafting

**DOI:** 10.1155/2016/1965720

**Published:** 2016-09-22

**Authors:** Mehmet Erol Can, Hasan Basri Çakmak, Gamze Dereli Can, Hatice Ünverdi, Yasin Toklu, Sema Hücemenoğlu

**Affiliations:** ^1^Department of Ophthalmology, Keçiören Training and Research Hospital, Ankara, Turkey; ^2^Department of Ophthalmology, Hacettepe University Faculty of Medicine, Ankara, Turkey; ^3^Department of Ophthalmology, Yıldırım Beyazıt University Faculty of Medicine, Ankara Atatürk Training and Research Hospital, Ankara, Turkey; ^4^Department of Pathology, Ankara Training and Research Hospital, Ankara, Turkey

## Abstract

*Purpose*. To investigate the effect of platelet-rich fibrin (PRF) membrane on wound healing.* Methods*. Twenty-four right eyes of 24 New Zealand rabbits equally divided into 2 groups for the study design. After the creation of 5 × 5 mm conjunctival damage, it was secured with PRF membrane, which was generated from the rabbit's whole blood samples in PRF membrane group, whereas damage was left unsutured in the control group. Three animals were sacrificed in each group on the 1st, 3rd, 7th, and 28th postoperative days. Immunohistochemical (IHC) stainings and biomicroscopic evaluation were performed and compared between groups.* Results*. PRF membrane generated significant expressions of vascular endothelial growth factor (VEGF), transforming growth factor-beta (TGF-*β*), and platelet-derived growth factor (PDGF) in the early postoperative period. However, the IHC evaluation allowed showing the excessive staining at day 28, in control group. Biomicroscopic evaluation revealed complete epithelialization in PRF membrane group, but none of the cases showed complete healing in the control group.* Conclusions*. This experimental study showed us the beneficial effects of the PRF membrane on conjunctival healing. Besides its chemical effects, it provides mechanical support as a scaffold for the migrating cells that are important for ocular surface regeneration. These overall results encourage us to apply autologous PRF membrane as a growth factor-enriched endogenous scaffold for ocular surface reconstruction.

## 1. Introduction

The conjunctiva, as an integral part of the ocular surface, has to be kept healthy and free of various disease processes that cause disruption in the integrity and function of the ocular surface. Minor conjunctival defects can be closed by primary intent; however, in the case of large tissue defects, the need for alternative covering materials for tension-free conjunctival closure is inevitable. Restoration of the ocular surface poses challenges following removal of lesions like pterygium, tumor, symblepharon, or conjunctivochalasis. In addition, complications resulting from a sequel of acute chemical burn or conjunctival scarring due to mucous membrane disorders, such as ocular cicatricial pemphigoid, Stevens-Johnson syndrome (SJS), or toxic epidermal necrolysis (TEN), necessitate conjunctival reconstruction, which requires large amounts of tissue replacement.

Human amniotic membrane is one of the most common biomaterials for ocular surface reconstruction in which condition the tissue defect is outsized. However, it has some disadvantages such as the risk of disease transmission, limited transparency, variable and unstable quality, and low mechanical strength. Other tissue substitutes include conjunctival autografts, oral mucosal grafts, nasal mucosal grafts, or* in vitro* limbal/mucous epithelial cell expansions employed with varying reported success rates [1–4]. All of these methods have drawbacks due to their need for more complex surgery with intrinsic complications, thereby preventing optimal treatment success.

Platelet-rich fibrin (PRF) membrane is a second-generation platelet concentrate which was first developed for oral and maxillofacial applications by the French Choukroun et al. in 2006 [5, 6].

Many growth factors including platelet-derived growth factor (PDGF), vascular endothelial growth factor (VEGF), and transforming growth factor-beta (TGF-*β*), which are released by PRF membrane during a period of at least 7 days and up to 28 days, and matrix proteins such as thrombospondin-1, fibronectin, and vitronectin, which have key roles in hemostasis and wound healing, exist in the PRF membrane [7, 8]. Furthermore, PRF membrane provides mechanical support as a scaffold for the cell proliferation, differentiation, and migration which are important for ocular surface regeneration [9]. Several clinical applications of PRF membrane have been described in oral surgery [5, 10, 11], periodontal regeneration surgery [12–18], treatment of meniscus tearing [19], treatment of chronic lower-extremity ulcers [20], ear-nose-throat procedures [21], plastic surgery [22–24], and ophthalmic surgery [25]. The combination of mechanical and chemotactic support of autologous PRF membrane makes it suitable for reconstruction, improvement, and/or maintenance of the tissue function and might offer many potential clinical and biotechnological advantages for tissue engineering applications in ophthalmology.

The purpose of this study was to evaluate the effects of autologous PRF membrane on rabbit conjunctival wound healing.

## 2. Methods

This was a prospective experimental animal study.

### 2.1. Experimental Animals

A total of 24 adult female New Zealand white rabbits aged between 12 and 30 weeks, weighing between 3000 and 3500 g at baseline, were used in this study. All animals were transferred from the Center of Refik Saydam Hıfzıssıhha, Ankara, Turkey, to Gazi University Animal Experiments Laboratory, Ankara, Turkey, 10 days prior to the study. The animals were individually maintained in a standard cage condition of a purpose-designed room for experimental animals and exposed daily to 12-hour-light/12-hour-night cycle with free access to a standard laboratory diet. All experimental methods and animal care procedures adhered to the Statement for the Use of Animals in Ophthalmic and Vision Research and were approved and monitored by the Institutional Animal Care and Use Committee at Legacy Health.

### 2.2. Anesthesia

During the surgical procedure, animals were anesthetized using an injection of 50 mg/kg ketamine (Brema-Ketamin 10%; Bremer Pharma, Germany) and 5 mg/kg xylazine (Alfazyne 2%; Alfasan International BV, Netherlands). After the surgery, all animals were injected with 3 mg/kg ketoprofen (Rifen 1%; Richter Pharma AG, Austria) for analgesia.

### 2.3. Experimental Design

**Figure 1:**
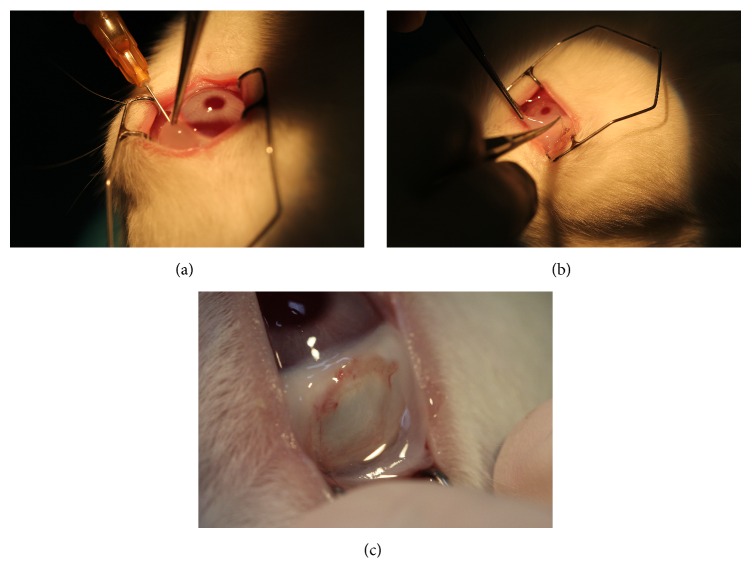
(a) Injection of BSS® into the subconjunctival space for dissection. (b) The excision of conjunctiva and Tenon's capsule with Westcott scissors. (c) A 5 × 5 mm square shaped tissue defect in the rabbit eye.

Unilateral (right eye) conjunctival damage in all rabbits was modeled by excision of the temporal side of the interpalpebral bulbar conjunctiva using an operating microscope. After the injection of 0.5 mL balanced salt solution (BSS) into the subconjunctival space (Figure 1(a)), approximately 5 × 5 mm square shaped conjunctiva and Tenon tissues were excised with Westcott scissors from a distance of 3 mm from the limbus (Figure 1(b)).

All rabbits were equally divided into 2 groups: the PRF membrane group (*n* = 12), in which the defect was secured with PRF membrane prepared from the rabbits' own whole blood samples (Figure 1(c)), and the control group (*n* = 12), in which no further procedure was done after the excision.

### 2.4. Preparation and Application of PRF Membrane

**Figure 2:**
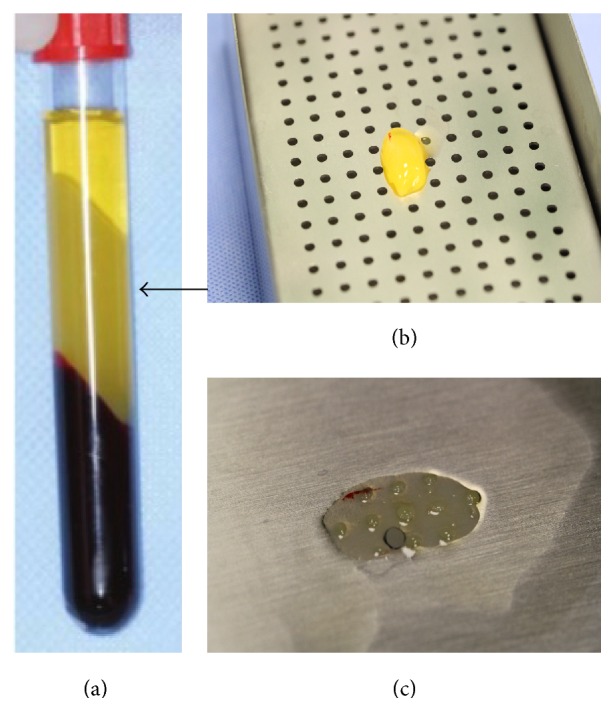
(a) After centrifugation, a fibrin clot (arrow) was placed between the acellular plasma layer at the top and the red corpuscles at the bottom of the tube. (b) Removal of the PRF clot from the tube using forceps. (c) PRF membrane obtained by compressing the PRF clot with PRF membrane box.

**Figure 3:**
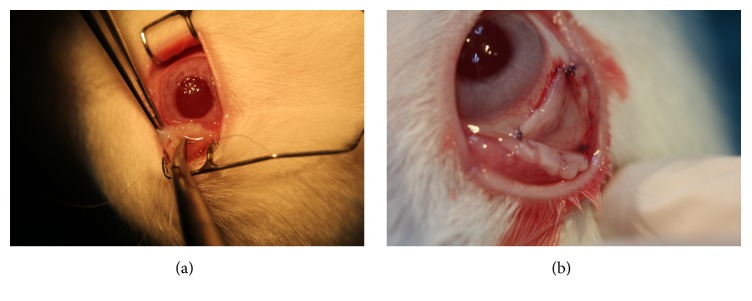
(a) PRF membrane was placed on the bare sclera and secured with 7-0 absorbable suture. (b) The immobilization of the PRF membrane over the defective zone.

To prepare the autologous PRF membrane, 5 mL fresh blood sample was drawn from the femoral vein of the rabbit and collected into a glass-coated tube without an anticoagulant under general anesthesia. Samples were immediately centrifuged at 2700 rpm (approximately 400 ×g) for 12 minutes using a table centrifuge system (Hettich EBA-20; Hettich Holding GmbH & Co. oHG, Germany). The fibrin clots were concentrated between the red blood cell corpuscles at the bottom of the centrifuge tubes and the acellular plasma, called platelet-poor plasma (PPP), at the top of the tubes (Figure 2(a)). PPP was then collected by pipetting the supernatant of the centrifuged blood sample. After the removal of PPP, fibrin clots were mechanically separated from the red blood cells with forceps (Figure 2(b)) and gently compressed using a custom-made PRF membrane box (PRF box; Medisoft Medikal, Turkey) to drain the remaining fluid (Figure 2(c)). Subsequently, PRF membrane was placed on the bare sclera and secured to the surrounding conjunctiva with 7/0 absorbable suture material (DLZ-6.4-200 FSSB, Germany) (Figure 3(a)). In order to immobilize the PRF membrane, 3 or 4 bites were placed with 7/0 vicryl interruptedly, 2 of them at the superior and the inferior limbal regions (Figure 3(b)). Postoperatively, moxifloxacin 0.5% (Vigamox; Alcon Lab, Texas) was instilled 4 times daily up to 10 days. Throughout the follow-up period, complications such as secondary infection, scleral necrosis, symblepharon, or any retraction formation in the fornices or eyelids were not observed.

### 2.5. Process of Enucleation and Tissue Collection

Three rabbits per group were sacrificed under general anesthesia by intravenous injection of 2 cc xylazine (Alfazyne 2%; Alfasan International BV, Netherlands) and the right eyes were enucleated on days 1, 3, 7, and 28 after surgery for histopathological evaluation.

### 2.6. The Preparation of Histology Slides and Grading of the Staining Pattern

The enucleated eyes of the rabbits in each group were fixed in 10% buffered formalin to prevent tissue autolysis and putrefaction for 24 hours at room temperature and embedded in paraffin. Four-micrometer-thick radial sections (3 or 4 slices) were taken from the paraffin embedding which contains the region between primary and defective tissue zones with a microtome, and sections were stained with hematoxylin and eosin (H&E). Inflammation, vascular proliferation, and fibrosis were evaluated considering the prevalence and severity of inflammatory cells, new vessels, and fibroblasts in tissue specimens in microscopic evaluation of 40x high power field (HPF) [26]. Grade 0 indicated that there was no inflammation, vascular proliferation, or fibrosis. Grade 1 demonstrated mild inflammation (<50 inflammatory cells in 40x HPF), mild vascular proliferation (<5 vessels in 40x HPF), and mild fibrosis. Finally, Grade 2 represented moderate to severe inflammation (>50 inflammatory cells in 40x HPF), moderate to severe vascular proliferation (>5 vessels in 40x HPF), and moderate to severe fibrosis.

The conjunctival sections were then mounted on poly-L-lysine coated slides and their controls were immunostained using Leica Bond Max (Leica; Wetzlar, Germany) automated immunostainer for VEGF, TGF-*β*, PDGF, and alpha-smooth muscle antigen (*α*-SMA) after antigen retrieval. The control tissues included small intestine for VEGF, breast carcinoma for TGF-*β*, and prostate adenocarcinoma for PDGF. Antibody detection was performed using a biotinylated secondary antibody and 3,3′-diaminobenzidine, and slides were observed with Olympus Imager BX51, DP25 (Olympus Optical, Tokyo, Japan) microscope. PDGF receptor (1 : 100 dilution; Abcam, Cambridge, MA), VEGF receptor (1 : 100 dilution; Abcam, Cambridge, MA), and TGF-*β* receptor (1 : 100 dilution; Novocastra Laboratories, Ltd., Newcastle upon Tyne, UK) were obtained from Ser-Med (Ankara, Turkey).

In order to analyse the antibody expressions, immunohistochemical staining patterns were scored with the staining intensity and morphology, a combination of qualitative and quantitative information, by the same examiner [26]. Grades 0, 1, and 2 indicated no staining, minimal staining, and severe staining, respectively. To avoid inaccuracy of the comparison of the staining intensity, all the photographs were taken with the same microscope with the same settings.

## 3. Results

### 3.1. Biomicroscopic Evaluation

**Figure 4:**
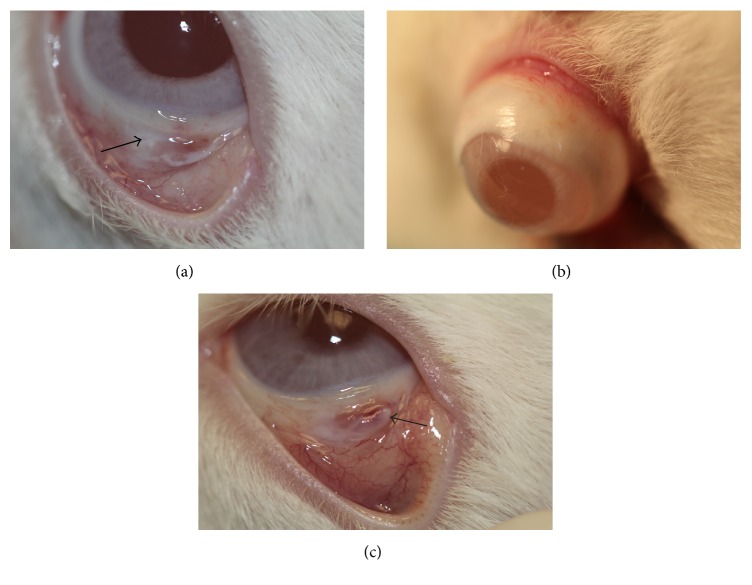
(a) Almost all of the defective zone reepithelialized on day 7 in the PRF membrane group. PRF membrane was integrated with the surrounding conjunctiva (arrow). (b) Conjunctival healing was completed on day 28 in the PRF membrane group without any complications. (c) The conjunctival defect (arrow) was persistent with mild hemorrhage on day 28 in the control group.

Biomicroscopic evaluation revealed reepithelialization of the bare sclera on the 1st week in the remaining 6 rabbits of the PRF membrane group (Figure 4(a)). Smooth transition was observed between the reepithelialized region and the primary conjunctival tissue without any ridge formation that might have caused irritation in the postoperative period. Throughout the follow-up period, complications such as secondary infection, scleral necrosis, symblepharon, or any retraction formation of the fornices or eyelids were not observed (Figure 4(b)). However, bare sclera was proceeded throughout the study period in the control group with mild to moderate hemorrhage and secretion formation indicated poor healing (Figure 4(c)).

### 3.2. H&E Staining

**Figure 5:**
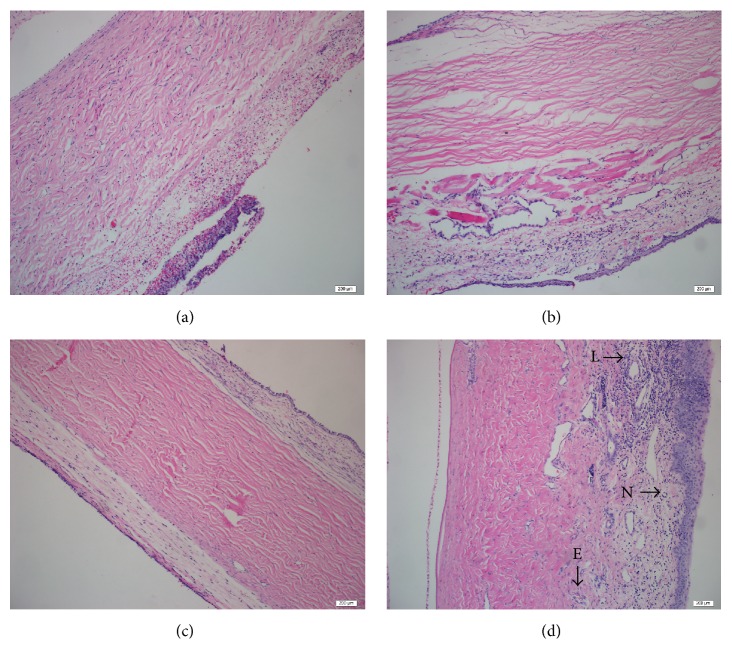
Comparison of the staining intensity of H&E. (a) Severe inflammatory reaction on the 1st day in PRF membrane specimens (×200). (b) Mild inflammation seen on the 1st day in the control group (×200). (c) Conjunctival tissue formation with normal histomorphology seen on day 28 in the PRF membrane group (×200). (d) Mixed cellular inflammation contains neutrophil leucocytes (N), lymphocytes (L), and eosinophils (E) seen on day 28 in the control group (×200).

All tissue specimens stained with H&E were examined to evaluate 3 important aspects of wound healing: inflammation, vascular proliferation, and fibrosis. Rabbits in the PRF membrane group showed major differences for inflammation when their condition was compared to rabbits in the control group. There was a severe inflammatory reaction on the 1st day in PRF membrane specimens. In the following days, for day 3 and day 7, the intensity of the inflammatory reaction began to be alleviated and ended on the 28th day. However, moderate inflammatory reaction was observed in the control group, in contrast to severe reaction in the PRF membrane group on the 1st day. Additionally, on day 3 and day 7, inflammation became more pronounced and continued even in the 28th-day specimens in the control group. As Figure 5 shows, there was a significant difference between groups in terms of inflammatory reactions.

Furthermore, there was a significant difference between the groups when the specimens were examined for vascular proliferation. There was minimal vascular proliferation in 3rd- and 7th-day specimens in the PRF membrane group. On the other hand, in the control group, moderate vascular proliferation was observed on the 1st week. The difference between groups became more apparent in the specimens by the 28th day. No vascular proliferation remained in the PRF membrane group, whereas signs of vascular proliferation persisted in the control group.

Additionally, significant differences have been found in certain areas between groups when the specimens were evaluated for fibrosis formation. In the PRF membrane group, mild fibrosis was observed in the 7th-day specimens. By contrast, in the control group, preliminary signs of pronounced fibrosis appeared first in the 7th-day specimens, resulting in significant fibrosis by the 28th day.

The results of the H&E staining are shown in Table 1.

### 3.3. Immunohistochemistry

#### 3.3.1. *α*-SMA

**Figure 6:**
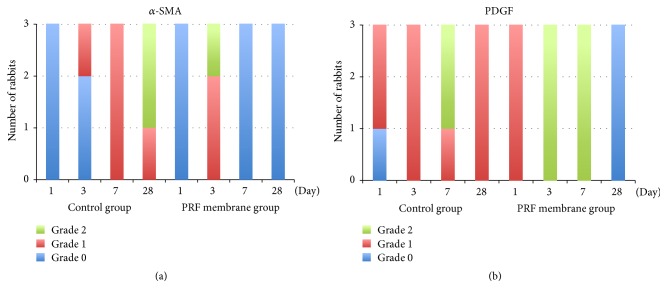
Comparison of the staining intensity of *α*-SMA and PDGF. (a) Histogram of the *α*-SMA staining. (b) Histogram of the PDGF staining.

Figure 6(a), showing *α*-SMA staining intensity, illustrates the main differences between groups. In the PRF membrane group, *α*-SMA staining was seen only in 2 cases on day 3. On the other hand, in the control group, the *α*-SMA staining started on day 3 and increased by the 7th day. The difference between PRF membrane and control groups became more striking on day 28. By that time, a marked *α*-SMA expression was observed in the control group, contrasting with a negative staining in all specimens in the PRF membrane group.

#### 3.3.2. PDGF

**Figure 7:**
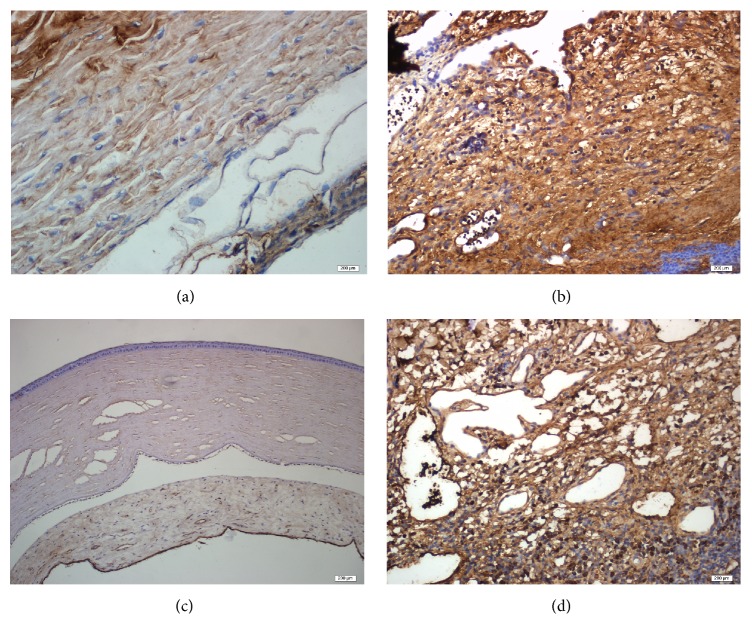
Immunohistochemical analysis of PDGF and TGF-*β*. (a) Grade 0 staining of PDGF was observed on day 28 in the PRF membrane group. (b) Grade 1 staining of PDGF was observed on day 28 in the control group. (c) Grade 0 staining of TGF-*β* was observed on day 28 in the PRF membrane group. (d) Grade 1 staining of TGF-*β* was observed on day 28 in the control group.

PDGF expressions were significantly different between groups. As Figure 6(b) shows, both on the 3rd and on the 7th days, PDGF expression was more pronounced in the PRF membrane group. On day 28, the PDGF expression could not be detected in the PRF membrane group, while in the control group there was Grade 1 staining in all specimens (Figures 7(a) and 7(b)).

#### 3.3.3. TGF-*β*

**Figure 8:**
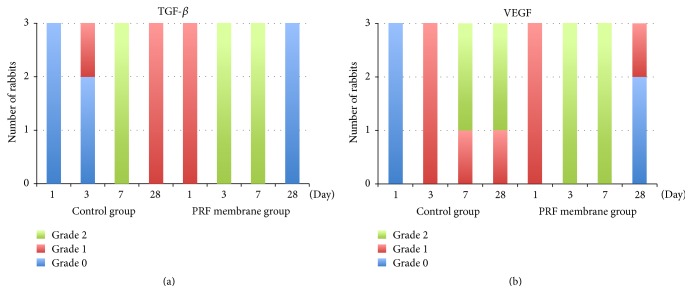
Comparison of the staining intensity of TGF-*β* and VEGF. (a) Histogram of the TGF-*β* staining. (b) Histogram of the VEGF staining.

Again, the expression of TGF-*β* began earlier, and its expression was more marked in the PRF membrane group. Interestingly, in the 28th-day specimens, there was no TGF-*β* staining in the PRF membrane group, which was in contrast to the Grade 1 staining seen in the control group (Figures 7(c) and 7(d)). The difference between groups with regard to TGF-*β* expression was similar to the difference in PDGF expression. This similarity was shown clearly in Figure 8(a).

#### 3.3.4. VEGF

The VEGF expression in the PRF membrane group was different from the control group in a number of important ways. In the PRF membrane group, even early specimens from the 1st day depicted moderate VEGF expression, and this expression became maximal on day 3 and stayed in the same level on day 7 (Figure 8(b)). Compared with the PRF membrane group, VEGF expression started later (on day 3) in the control group. Most importantly, examination of the 28th-day specimens showed significant differences between groups. The VEGF staining was continued in the control group, whereas, in the PRF membrane group, only 1 specimen was stained as Grade 1.

The results of the immunohistochemistry staining are summarized in Table 2.

## 4. Discussion

Conjunctival wound healing is a complex process in which a variety of cytokines, growth factors, and proteases interact to regulate the key phases of the healing.

In general, conjunctival wound healing is similar to wound healing in normal tissue, which is a complex and a dynamic three-phase process. These phases are the inflammatory phase, the proliferative phase, and the remodeling phase [27–29].

The inflammatory phase of wound healing is characterized by the infiltration of neutrophils and monocytes into the wound area. At this stage, plasma proteins and extracellular matrix fragments are released into the wound area due to injury of the connective tissue and blood vessels. Furthermore, platelet aggregation and hemostasis cascade have been activated at the wound site. The hemostatic plugs mainly consist of fibrinogen. The fibrin molecule is the final product, which is derived from fibrinogen, and provides support like a scaffold material over the wound area. Furthermore, growth factors, proteases, and metabolites of arachidonic acids are released as a result of the activated platelets and coagulation cascade [28]. The main factors released from the platelets are PDGF, VEGF, TGF-*α*, and TGF-*β* [5, 6]. Releasing growth factors leads to cell migration and proliferation in the healing site. After this stage, the proliferation of macrophages and fibroblast cells occurs in the proliferation phase. In addition, intensive synthesis of collagen, fibronectin, and proteoglycan helps the formation of angiogenesis and granulation tissue. During the remodeling phase, the degradation of extracellular matrix occurs, tensile strength of the tissue increases, and the vascularity and cellularity decrease. This period can last up to 2 weeks to several months.

The PDGF and particularly TGF-*β* are the key components of the fibrotic response in wound healing. TGF-*β* stimulates the migration of fibroblasts that synthesize the extracellular matrix. As a result, TGF-*β* is involved in the mechanism of most diseases arising from excessive fibrosis, like glaucoma and proliferative diabetic retinopathy [30, 31]. The PDGF is a major mitogen for connective tissue cells and it plays an important role in wound healing [32, 33]. It acts on several cell types involved in the wound healing phases. It stimulates fibroblasts, smooth muscle cells, neutrophils, and macrophages [34]. The PDGF also stimulates the production of extracellular matrix molecules like fibronectin, collagen, proteoglycan, and hyaluronic acid [35–38]. At the early stage of the wound healing, PDGF is released by platelets and secreted by activated macrophages, thrombin-stimulated endothelial cells, smooth muscle cells of damaged arteries, and activated fibroblasts [39–42]. As a result of releasing PDGF, reepithelialization, angiogenesis, and extracellular matrix deposition have occurred at the healing site [35]. In the remodeling phase, PDGF stimulates the production and secretion of collagenase by fibroblasts [43]. Therefore, the overactivity of PDGF is related to scarring and fibrosis [33, 44].

Platelet-rich plasma (PRP) and its products have been used in dentistry and maxillofacial surgery for tissue regeneration for many years. PRF, described by Choukroun et al. [5], is a second-generation platelet concentrate, consisting of many growth factors and cytokines which play a key role in hemostasis and wound healing. These growth factors are known to promote cell proliferation, differentiation, migration, and matrix synthesis by binding to specific cell surface receptors [5, 29]. Furthermore, PRF clots trap stem cells, which are circulating in the peripheral blood and contribute to wound healing. The PRF membrane, the resistant fibrin membrane, can be easily obtained by pressing the PRF clot which is localized in the middle part of the centrifuge tube between the PPP and red blood cell layer with a simple press machine.

In this prospective study, we clearly demonstrated that the influence of PRF membrane on conjunctival wound healing is supportive due to its biological and physical properties. In the earlier phase of wound healing, the PRF membrane appeared to promote the release of growth factors in higher amounts than the normal wound healing cycle. However, the expression of growth factors started to diminish by the postoperative 7th day while the PRF membrane was disappearing. Furthermore, PRF membrane provided mechanical support to the migrating conjunctival cells as a scaffold. Consequently, we did not observe any scar formation or inflammation in H&E staining in the PRF membrane group on day 28. Hence, it could be conceivably hypothesized that PRF membrane modified the TGF-*β* expression, suppressed its overexpression, and prevented scar formation. In addition, biomicroscopic evaluation revealed that smooth new conjunctival tissue without any roughness was formed over the defective zone in PRF membrane group. However, in the control group, mild immunohistochemical staining intensity was observed on day 3 and day 7, in contrast with the severe intensity on day 28. As a result, the wound healing process had been sustained, resulting in continuing fibrosis and inflammation even on the 28th day. This suggested that the scaffold function of the PRF membrane and excessive and early release of the growth factors from the PRF membrane might have a positive effect on conjunctival healing.

PRF membrane offers many advantages compared to other methods that have been used for conjunctivoplasty. First, the architecture of the PRF membrane facilitates the cellular proliferation, differentiation, and especially migration and provides an important temporary mechanical support to the growing cells. Alongside the conjunctival epithelial cells, the endothelial cells that are necessary for neoangiogenesis, vascularization, and survival of the graft could easily proliferate and migrate on/into this membrane for the regeneration of the defective zone. Second, the platelet cytokines are released gradually as the fibrin matrix gets resorbed, thus creating a convenient process of healing. Lastly, the presence of leukocytes and cytokines in the fibrin network can play a significant role in the self-regulation of the inflammatory and infectious phenomenon within the grafted material [14, 45]. All these features make PRF membrane a feeder layer for conjunctival epithelial cells which is actually used for* in vitro* cell cultivation in cell and tissue engineering applications. Additionally, the gradual release of the growth factors mimics the controlled release mechanism of the biosignals that researchers try to create for tissue engineering applications.

Besides these theoretical advantages, the ease of obtainment and implementation of the PRF membrane makes it suitable when compared to other methods that have been used for clinical applications. Conjunctival autograft, the conventional method for conjunctivoplasty, is the most popular option for the treatment of conjunctival tissue defects caused by the excision of pterygium, tumor, or symblepharon or conjunctival cicatrization caused by SJS or TEN. But it cannot be performed in cases with large defects or cases that need future surgery for glaucoma. Furthermore, conjunctival autograft might cause limbal stem cell deficiency that is important for the ocular surface health. The other option for the reconstruction of the ocular surface is amniotic membrane transplantation (AMT), which is considered one of the major new developments in ocular surface surgery. Similar to PRF membrane, the amniotic membrane promotes epithelialization by acting as a temporary basement membrane and releases growth factors such as epidermal growth factor and keratocyte growth factor that are important for the healing. Further, it has anti-inflammatory and antiscarring effects by the inhibition of TGF-*β* signaling in the chronic term. However, the preparation of amniotic membrane is complicated and rather expensive. In addition, strong tissue banking expertise is crucial to prevent inadvertent complications. The contagion risk of some serious pathogens always exists and cannot be excluded even in the presence of very strict procedures and measures. Furthermore, amniotic membrane is a natural but an allogenic matrix, and immunologic response to allografts is the major concern. However, PRF membrane is autologous and is prepared from the patient's own blood samples and poses no immunological rejection risk to the grafted tissue. In addition, PRF membrane is cost-effective and carries no risk of allergic reactions. The preparation of PRF membrane is simple and practical. It can be carried out with few instruments. This process does not require complicated and expensive equipment. Moreover, blood samples could easily be taken, and the PRF membrane could easily be prepared in a short amount of time after centrifuging the blood samples.

To the best of our knowledge, this is the first animal study investigating the role of PRF membrane in conjunctival wound healing. Although the results are encouraging, the small number of rabbits and the short follow-up time for the evaluation of the effectiveness of the PRF membrane are the main limitations of our study. Furthermore, some of the parameters, such as imaging wound area and intensity of scar formation, need to be optimized.

In conclusion, PRF membrane appears to be a novel treatment alternative to other treatment modalities which are currently employed for conjunctivoplasty. Intrinsic trophic substances and the micro- and macroarchitecture of the PRF membrane make it an ideal substrate for reconstruction of the ocular surface. These results may encourage us to use PRF membrane for debilitating ocular surface disorders with a pronounced inflammatory reaction resulting in severe vision loss.

## Figures and Tables

**Table 1:** The comparison of hematoxylin and eosin staining.

Day	PRF membrane group			Control group		
	Inflammation	Vascular proliferation	Fibrosis	Inflammation	Vascular proliferation	Fibrosis
1	++	+	−	+	+	−
3	++	++	−	++	++	−
7	++	+	+	++	++	+
28	−	+	−	++	+	++

−: Grade 0, no inflammation, vascular proliferation, or fibrosis.

+: Grade 1, mild inflammation (<50 inflammatory cells) (×40), mild vascular proliferation (<5 vessels) (×40), and mild fibrosis.

++: Grade 2, moderate to severe inflammation (>50 inflammatory cells) (×40), moderate to severe vascular proliferation (>5 vessels) (×40), and moderate to severe fibrosis.

PRF: platelet-rich fibrin.

**Table 2:** The comparison of the immunohistochemical staining.

Day	PRF membrane group			Control group		
	VEGF	PGDF	TGF-*β*	VEGF	PGDF	TGF-*β*
1	+	+	+	−	+	−
3	++	++	++	+	++	+
7	++	++	++	++	++	++
28	−	−	−	++	+	+

−: Grade 0, no staining.

+: Grade 1, minimal staining.

++: Grade 2, moderate to severe staining.

PRF: platelet-rich fibrin; VEGF: vascular endothelial growth factor; PDGF: platelet-derived growth factor; TGF-*β*: transforming growth factor-beta.
